# Urinary 2,5-hexanedione excretion in cryptogenic polyneuropathy compared to the general Swedish population

**DOI:** 10.1186/1745-6673-8-21

**Published:** 2013-07-30

**Authors:** Bodil Persson, Magnus Vrethem, Nicola Murgia, Jonas Lindh, Anna-Lena Hällsten, Mats Fredrikson, Martin Tondel

**Affiliations:** 1 Division of Occupational and Environmental Medicine, Department of Laboratory Medicine, Skåne University Hospital, Lund, Sweden; 2 Division of Neurology and Neurophysiology, Department of Clinical and Experimental Medicine, Faculty of Health Sciences, Linköping University, Linköping, Sweden; 3 Department of Neurology and Neurophysiology, County Council of Östergötland, Linköping, Sweden; 4 Section of Occupational Medicine, Respiratory Diseases and Toxicology, University of Perugia, Perugia, Italy; 5 Department of Internal Medicine, Section of Neurology, Ryhov County Hospital, Jönköping, Sweden; 6 Division of Occupational and Environmental Medicine, Department of Clinical and Experimental Medicine, Faculty of Health Sciences, Linköping University; 7 Department of Occupational and Environmental Medicine, County Council of Östergötland, Linköping, Sweden; 8 Occupational and Environmental Medicine, Department of Medical Sciences, Uppsala University, Uppsala, Sweden

**Keywords:** Polyneuropathy, Cryptogenic, Urine, 2,5-hexanedione, General population, Sweden, Occupational exposure

## Abstract

**Background:**

2,5-hexanedione (2,5-HD) is the main neurotoxic metabolite of methyl-n-butyl ketone (MBK) and n-hexane, and known to cause polyneuropathy. The aim of our study was to compare the urinary levels of 2,5-HD between cases with cryptogenic polyneuropathy and the general Swedish population, and to elucidate the role of certain external factors.

**Methods:**

Morning urine samples were collected from 114 cases with cryptogenic polyneuropathy (77 men and 37 women) and 227 referents (110 men and 117 women) randomly selected from the population registry. None had any current occupational exposure to n-hexane or MBK. The urine samples were analysed by a gas chromatographic method based on acidic hydrolysis.

**Results:**

Cases had statistically higher urinary levels of 2,5-HD (0.48 mg/L) than the general population (0.41 mg/L) and men higher excretion than women (0.48 mg/L and 0.38 mg/L, respectively). There was no difference in 2,5-HD levels between current smokers and non-smokers. Occupational exposure to xylene, alcohol consumption and ever exposed to general anaesthesia were associated with lower excretion in men while for occupational exposure to nitrous oxide in women higher excretion was seen. Higher excretion of 2,5 HD was inversely related to increasing age.

**Conclusions:**

Significantly higher levels of urinary 2,5-HD were seen in men and cryptogenic polyneuropathy cases seemingly unexposed to n-hexane. Hypothetically, this might be due to either differences in metabolic patterns or some concealed exposure. The difference in means between cases and the general population is small and can therefore not allow any firm conclusions of the causality, however.

## Introduction

Polyneuropathy is a common disorder of the peripheral nervous system in middle and late adulthood. The aetiology is sometimes not clear, even after thorough medical examination, and the polyneuropathy is then referred to as cryptogenic. Studies suggest a role of occupational exposures in the aetiology of cryptogenic polyneuropathy. We have previously scrutinized clinical and neurophysiological characteristics of patients with cryptogenic polyneuropathy and investigated occupational determinants [1,2]. In a case-referent study increased risks were found in men for occupational exposure to sulphur dioxide, xylene, methyl ethyl ketone, and herbicides along with solvent exposure in leisure time, whereas in women increased risks for occupational exposure to lead, nitrous oxide, and insecticides were found [2].

The organic solvents methyl-n-buthyl ketone (MBK) and n-hexane are well known to cause polyneuropathy through the toxic metabolite 2,5-hexanedione (2,5-HD) [3,4]. MBK has even greater neurotoxic potential than n-hexane and furthermore another organic solvent, methyl-ethyl-ketone (MEK) is known to potentiate the neurotoxic effect [5,6]. MBK and n-hexane are used for adhesives or extraction. N-hexane is also a compound derived from cracking of petroleum. In Sweden the industrial use of these solvents has decreased dramatically the latest decades but n-hexane might still exist as contaminants in other solvents and in petrol for cars. Previously n-hexane was used in the extraction of vegetable oil for e.g. margarine production with a threshold limit value of 1 mg/kg applied by the National Food Agency in Sweden. As a consequence, there are various routes of n-hexane exposure to the human body (inhalation, dermal and oral) and it is therefore difficult to estimate the total exposure in the environment, because both occupational and life style factors can contribute.

The urinary metabolite 2,5-HD can be measured in individuals occupationally exposed to n-hexane or MBK and is regularly used for biological monitoring of workers exposed. 2,5-HD can be measured as free metabolite or as total 2,5-HD after acid hydrolysis. For monitoring of workers total 2,5-HD has been frequently used worldwide, but in 2011 the American Conference of Governmental Industrial Hygienists (ACGIH) set up a biological exposure index for free 2,5-HD in workers exposed to n-hexane or MBK of 0.4 mg/L [7].

Urinary 2,5-HD has not only been detected in workers with occupational exposure to n-hexane, but is also found in low concentrations in the general population (Table 1) [8-15]. The aim of this study was to compare the excretion of 2,5-HD between cases of cryptogenic polyneuropathy with no known occupational exposure to n-hexane and the general population, and to elucidate the influence of certain external factors.

**Table 1:** Urinary levels of 2,5-hexanedione (2,5-HD) in general populations

**Country**	**n (men: women)**	**2,5-HD mean ± SD mg/L**	**2,5-HD range mg/L**	**Method**^**1**^	**Reference**
Germany	8:4	0.45 ± 0.20	0.12-0.78	GC-MS	[8]
Italy	10	0.49 ± 0.14	0.32-0.64	GC-FID	[9]
Japan	55:0	1.47 ± 0.60	--	GC-FID	[10]
Japan	53:0	0.33 ± 0.47	--	GC-FID	[11]
Italy	26	0.56 ± ?	0.17-0.98	GC-MS	[12]
Italy	20:20	0.47 ± 0.21	0.10-1.00	GC-FID	[13]
Italy	22	0.44 ± 0.11	0.18-0.73	HPLC-F	[14]
Italy	60:63	--	0.08-0.95	GC-FID	[15]

*GC-MS* Gas Chromatography with Mass-Selective detection, *GC-FID* Gas Chromatography with Flame Ionization Detection, *HPLC-F* High Performance Liquid Chromatography-Fluorescence detection.

^1)^after acid hydrolysis.

## Methods

### Cases

The subjects in this study had previously been included in a case-referent study, encompassing 164 cases of cryptogenic polyneuropathy and 604 referents [2]. All subjects lived in Östergötland or Jönköping counties in the southeast of Sweden. Out of the 164 cases 114 (77 men and 37 women, mean age 70 years with a range of 43 to 88 years), could be included*,* 227 referents (110 men and 117 women, mean age 64 years with a range of 46 to 85 years) out of the 300 randomly selected referents from the same study were included for urine analysis of 2,5-HD since 50 cases and 73 referents declined to participate.

The cases of cryptogenic polyneuropathy were diagnosed in three departments of neurology (University Hospital in Linköping, Motala Hospital, and Ryhov County Hospital in Jönköping). The databases of the hospitals were searched for all outpatients between the ages of 40 and 79 years with a diagnosis of polyneuropathy. Two versions of the International Classification of Diseases (ICD) were used in the time period of the study, ICD-9 and ICD-10, in adopted Swedish versions. The diagnosis of cryptogenic polyneuropathy corresponds to the international classifications ICD-9 356.4, 356.9, 357.9 and ICD-10 G60.9, G61.9, G62.9, respectively. All the medical records were re-examined by two neurologists (JL, MV) to confirm the correct diagnoses. Diagnosis of polyneuropathy was based on clinical symptoms and findings and was defined as one or more typical symptoms (distal paresthesias, numbness, neurogenic pain, distal weakness, loss of distal sweating) and at least 2 of 3 clinical findings (distal deficit of sensation, reduced distal muscle strength, and impaired or lost deep tendon reflexes). We excluded patients with a mainly demyelinating polyneuropathy to rule out Chronic Inflammatory Demyelinating Polyneuropathy (CIDP) and other inflammatory neuropathies. Patients under 40 years of age were also excluded to avoid inclusion of hereditary forms of polyneuropathy that had not yet been diagnosed. Out of the 114 patients, 91 had been examined with nerve conduction velocity to further establish a diagnosis of axonal polyneuropathy and to exclude demyelinating polyneuropathy. The following laboratory investigations had to be performed and to be normal: haemoglobin, serum glucose, cobalamin, folate, and thyroid function. Exclusion criteria were also known diabetes, renal failure, alcohol abuse, cobalamin deficiency, or other malignant or systemic diseases known to be a possible cause of polyneuropathy.

### Exposure

Occupational and environmental exposure information was received from a postal questionnaire used in the case-referent study [2] and included questions regarding other factors of potential interest i.e. food habits, alcohol consumption and smoking habits not presented in the previous study. Average alcohol consumption during one month was categorized in the analysis as one group with and one group without regularly alcohol intake (wine, beer, spirits).

### Urine samples

Both cases and referents were asked to cast a morning urine sample, which was collected in a 10 ml polyethylene test tube. The test tube was frozen to −70 degrees Celsius. The concentration of the metabolite 2,5-HD was analysed by a gas chromatographic method based on acid hydrolysis as previously described [16]. The precision of the method was 3.9% inter-assay and 0.7% intra-assay and the detection limit was 0.1 mg/L. Five urinary samples, all referents, showed values below the detection limit (0.00, 0.03, 0.04, 0.08 and 0.09 mg/L) and were after consideration included in the statistical analysis. The 2,5-HD levels were not adjusted to the urine creatinine concentration since we aimed to compare our results for the Swedish population to other populations and most other studies were reported without such correction [8-15]. Moreover, as mentioned earlier, the urine samples were collected as morning samples, hence less sensitive to dilution.

Furthermore, a classification of various solvents with regard to n-hexane containing solvents and thereby the metabolic formation of 2,5-HD, was performed by two occupational physicians (B.P., M.T.) and one occupational hygienist (P.S.) independently. Organic solvents forming 2,5-HD were merged into a new variable i.e. methyl-n-buthyl ketone, petrol, Stoddard solvent, jet fuel and n-hexane (Table 2).

**Table 2:** Exposure variables included in different linear regression models with respect to urinary 2,5-HD levels

**Occupational exposure**		**Life style**	**Solvent exposure**
Model 1 Men	Model 1 Women	Model 2	Model 3
Sulphur dioxide	Lead	Born in Sweden	2,5-hexanedione solvents^1^
Xylene*	Nitrous oxide*	Current smoking	Nitrous oxide occupationally*^for women^
Methyl ethyl ketone	Insecticides	Fruit several times/week	Solvents in leisure time
Herbicides		Vegetables several times/week	General anaesthesia and surgery*^for men^
Solvents leisure time		Meat several times/week	
		Fish several times/week	
		Alcohol ≥2 times/month*^for men^	

^1^Methyl-n-buthyl ketone, petrol, Stoddard solvent, jet fuel, n-hexane. * p<0.05. Xylene, alcohol consumption and anaesthesia was associated with lower 2,5-HD urinary excretion in men. Nitrous oxide exposure was associated with higher 2,5-HD urinary excretion in women.

In all models case/referent status and year of birth was included and analysed separately by sex.

### Ethical approval

The ethics committee at the Faculty of Health Sciences at Linköping University has approved the study.

### Statistical methods

The Kolmogorov-Smirnov test showed that the urinary 2,5-HD levels could be regarded as normally distributed (p<0.1) and thus the student’s t-test could be applied in the univariate analyses performed to compare the differences in mean urinary 2,5-HD levels between cases and referents. A p-value of <0.05 was considered statistically significant. In order to investigate the influence of various factors on the urinary excretion of 2,5-HD we applied different linear regression models. Included in all models were case-referent status (to have cryptogenic polyneuropathy or not) and year of birth. All these linear regression analyses were performed separately for men and women. Analyses were performed using Stata (Statistical Software, release 8.0; Stata Corp., College Station, Tex., USA).

## Results

**Figure 1:**
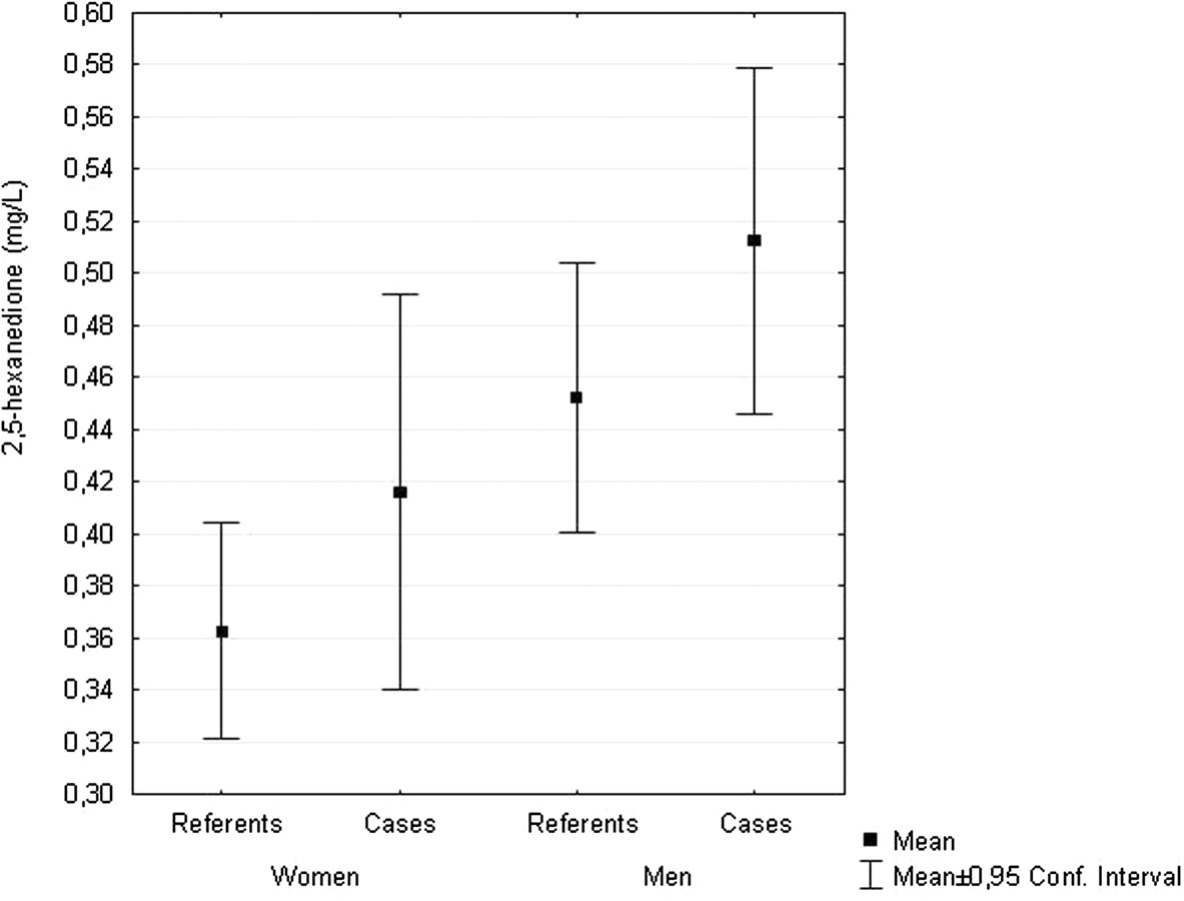
Urinary levels of 2,5-hexanedione (2,5-HD) in mg/L, mean and 95% confidence interval, in cases and referents and men and women.

**Figure 2:**
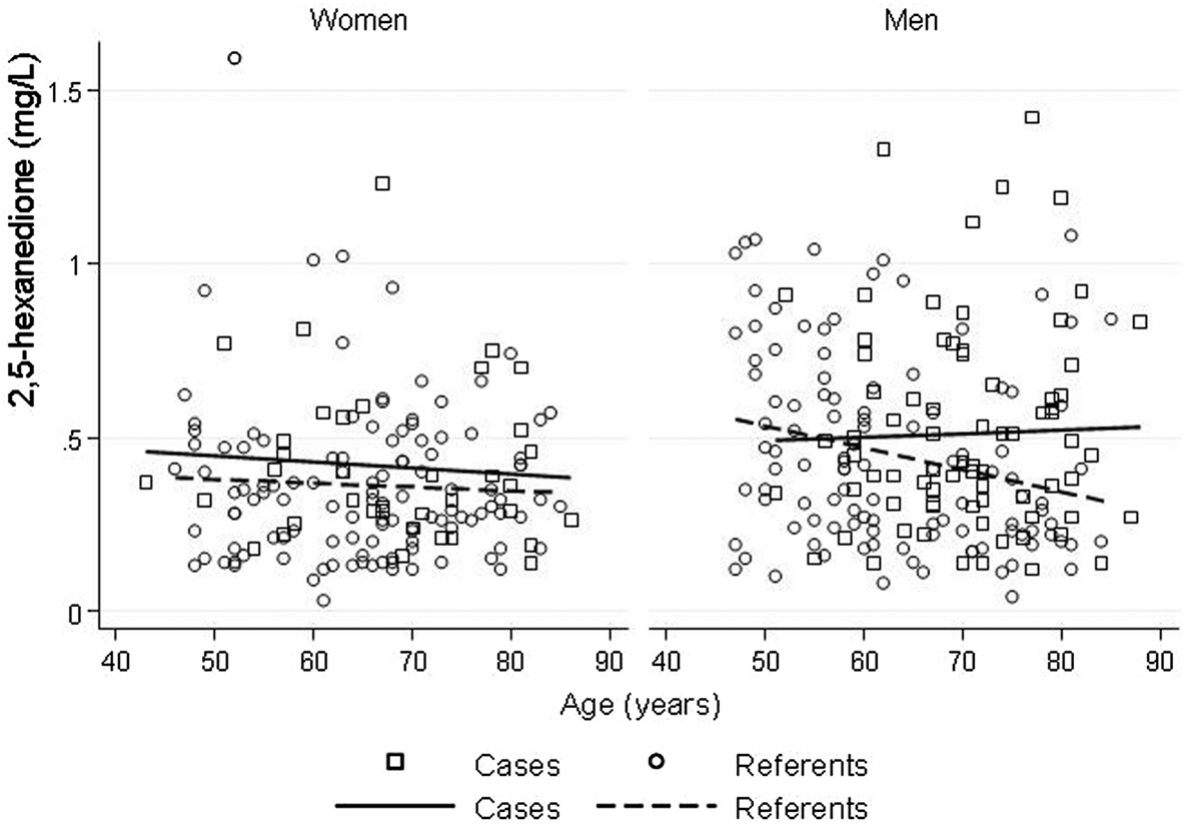
Urinary levels of 2,5-hexanedione (2,5-HD) in mg/L in men and women, and cases and referents, separately, in relation to age.

Cases had statistically significant higher excretion of 2,5-HD in urine than referents (mean 0.48 mg/L compared to 0.41 mg/L) and men had statistically significant higher excretion than women (mean 0.48 mg/L compared to 0.38 mg/L) (Table 3, Figure 1). Excretion of 2,5-HD was higher in men compared to women within both cases and referents, however only statistically significant for the larger group of referents (Table 3). Also the urinary levels were higher for cases than for referents of both sexes although not statistically significant (Table 3). The excretion of 2,5-HD was inversely related to increasing age only for male referents (Figure 2). There was no statistically significant difference in urinary 2,5-HD levels between current smokers and non-smokers. No men reported occupational exposure to nitrous oxide.

**Table 3:** Urinary excretion of 2,5-hexanedione (2,5-HD) in mg/L related to sex and case/referent status, age distribution and numbers of subjects included

**Status**	**n**	**Mean age (range)**	**2,5-HD±SD**	**p-value**	**Sex**	**n**	**2,5-HD±SD**	**p-value**
Case	114	70 (43–88)	0.48±0.28	0.01	Men	77	0.51±0.29	0.08
					Women	37	0.42±0.23	
Referent	227	64 (46–85)	0.41±0.25		Men	110	0.46±0.27	0.004
					Women	117	0.36±0.23	
**Sex**	**n**	**Mean age (range)**	**2,5-HD±SD**	**p-value**	**Status**	**n**	**2,5-HD±SD**	**p-value**
Men	187	66 (47–88)	0.48±0.28	0.0002	Case	77	0.51±0.29	0.22
					Referent	110	0.46±0.27	
Women	154	66 (43–86)	0.38±0.23		Case	37	0.42±0.23	0.21
					Referent	117	0.36±0.23	

In all the linear regression analyses case/referent status and year of birth were statistically significant variables for men, but not for women, with higher urinary 2,5-HD excretion for male cases and younger men.

Occupational exposures identified with logistic odds ratios >3.50, in the previous case-referent study [2] were included in the first model (i.e. for men: occupational exposure to sulphur dioxide, xylene, methyl ethyl ketone, herbicides and also solvent exposure in leisure time and for women: occupational exposure to lead, nitrous oxide and insecticides) (Table 2). In this model only occupational exposure to xylene in men was significant in relation to lower urinary 2,5-HD excretion levels (p= 0.03; regression coefficient −0.30; 95% confidence interval (CI) -0.57 to −0.03). For women only occupational exposure to nitrous oxide (p<0.05; regression coefficient 0.32; 95% CI 0.01 to 0.64) was significant, but with higher urinary 2,5-HD levels among the occupationally exposed.

Attributes in the second model included: born in Sweden, current smoking, food habits and alcohol consumption (Table 2). Among these life style factors in model 2 only alcohol consumption (p=0.01; regression coefficient −0.11; 95% CI −0.20 to −0.03) was statistically significant for men, with lower excretion among men with more frequent drinking. None of the variables in model 2 showed any significant results for women.

Included in the third model were various occupational solvent exposures, occupational exposure to nitrous oxide, organic solvent exposure in leisure time, and a history of surgery performed under general anaesthesia (Table 2). General anaesthesia (p=0.01; regression coefficient −0.11; 95% CI −0.19 to −0.02) was significantly associated with lower urinary 2,5-HD levels in men. In women higher excretion of urinary 2,5-HD was seen in those exposed to nitrous oxide (p<0.05; regression coefficient 0.33; 95% CI 0.01 to 0.65). Organic solvents metabolised to 2.5-HD showed no statistic significance in men or women.

## Discussion

Numerous studies have shown that 2,5-HD is a neurotoxic metabolite and causes polyneuropathy in occupational exposure to n-hexane [17,18]. Most studies, however, have been performed in industries with occupationally exposure to n-hexane [19-21] and less is known about the importance for development of neuropathy in presumably occupationally unexposed individuals.

In our study men with cryptogenic polyneuropathy had higher urinary excretion of 2,5-HD. Men showed consistently higher excretion than women in all analyses. We also found higher excretion of 2,5-HD inversely related to increasing age. Levels of urinary 2,5-HD in the general population were in accordance to previous published results, especially in European populations (Table 1). Exposure to xylene, alcohol, and general anaesthesia were associated with lower excretion in men, but for occupational exposure to nitrous oxide in women a higher excretion was seen. Xylene, alcohol and drugs used for general anaesthesia are all metabolised in the liver giving rise to a possible hepatic enzyme induction affecting the metabolism of n-hexane. In contrast, nitrous oxide is an inorganic gas with minimal or no metabolism in human tissues [4]. These facts might support the finding of reverse results between certain exposures and the urinary excretion. The differences in urinary 2,5-HD excretion between the sexes can also reflect gender variations in exposure.

Cases had no current occupational exposure to n-hexane, but micro exposure in the environment could not be excluded. If existing randomly among cases and referents, however, this would probably not explain the higher excretion in cases. We also do not have data on men having different micro exposure to n-hexane than women explaining the difference in urinary 2,5-HD excretion between the sexes. Another hypothesis is lipid peroxidation within the body resulting in formation of 2,5-HD, but no information on different metabolism between sexes, or for cases with polyneuropathy, is available.

The formation of 2,5-HD from n-hexane involves several genes and an association between the CYP2E1 gene might increase the susceptibility to develop n-hexane induced polyneuropathy [22]. Among our cases we found only two individuals with the gene expression CYP2E1 and we had no information about this gene among the referents. Therefore, we excluded these two cases and it did not change the mean values of 2.5-HD. Another gene involved in n-hexane metabolism is GSTT1. In a previous study, an increased risk for cryptogenic polyneuropathy was found in smokers with the gene GSTT1 null [23]. Furthermore, urinary 2,5-HD excretion is increased in smokers versus non-smokers [24]. In our study, however, we did not find any differences in 2,5-HD levels in urine between smokers and non-smokers.

In Parkinson’s disease poor metabolization of n-hexane has been proposed as a risk factor for the disease because of lower urinary 2,5-HD levels compared with controls [24]. In contrast to Parkinson’s disease, our cases of cryptogenic polyneuropathy had higher urinary 2,5-HD than the general population. Corroborating our results, however, the 2,5-HD levels inversely correlated to increasing age [24].

From the methodological point of view, even if ACGIH nowadays considers “free” 2,5-HD as biological exposure index for workers exposed to n-hexane, we had decided to use total 2,5-HD. As mentioned earlier and to allow comparisons with urinary concentrations, almost all previous studies considering concentrations of 2,5-HD in non-occupationally exposed populations have used total 2,5-HD. Moreover, when the study was performed ACGIH was still using total 2,5-HD as a biological exposure index in workers exposed to n-hexane and MBK [25]. The choice of “total” or “free” 2,5-HD as index of exposure is under debate and still controversial [26].

## Conclusions

To our knowledge this is the first study investigating urinary 2,5-HD levels in cases with cryptogenic polyneuropathy compared to the general population. We found significantly higher levels for the cases. Even if the differences in 2,5-HD excretion were small our study suggests that some external exposures, along with sex and age, could influence the excretion of 2,5-HD in subjects occupationally unexposed to n-hexane and MBK. On the other hand, it might also reflect a different metabolic pattern among the cases compared to the general population. The slight differences do not allow any conclusions about the aetiological role even though n-hexane exposure is a well-known cause of polyneuropathy.

## Competing interests

There are no financial conflicts of interest related to the material presented.

## Authors’ contributions

BP, MV, JL and MT were involved in the conception and design of the study, data collection, data analysis and interpretation, manuscript writing and final approval of the manuscript. NM was responsible for the urine analyses and their interpretation, manuscript writing and final approval of the manuscript. ALH was involved in the interpretation of the results, manuscript writing and final approval of the manuscript. MF was involved in the interpretation of the results, statistical analyses and manuscript writing and final approval of the manuscript.
